# Predicting Chinese father involvement: Parental role beliefs, fathering self-efficacy and maternal gatekeeping

**DOI:** 10.3389/fpsyg.2022.1066876

**Published:** 2022-12-16

**Authors:** Yang Liu, Divna M. Haslam, Cassandra K. Dittman, Mingchun Guo, Alina Morawska

**Affiliations:** ^1^School of Psychology, Fujian Normal University, Fuzhou, China; ^2^Parenting and Family Support Centre, School of Psychology, The University of Queensland, Brisbane, QLD, Australia; ^3^School of Law, Queensland University of Technology, Brisbane, QLD, Australia; ^4^School of Health, Medical and Applied Sciences, Central Queensland University, Bundaberg, QLD, Australia

**Keywords:** father involvement, Chinese, beliefs, maternal gatekeeping, fathering self-efficacy

## Abstract

**Introduction:**

Despite the well-established importance of positive father involvement for child adjustment, father involvement tends to be much lower compared to mother involvement. Furthermore, there are few empirical studies on Chinese fathers and their involvement in parenting. Given the importance of father involvement, it is necessary to examine the factors that may facilitate or hinder Chinese father involvement in parenting.

**Methods:**

This study used survey methodology to examine the predictors of Chinese father involvement with their preschoolers. The sample consisted of 609 Chinese parent dyads in Mainland China.

**Results:**

Fathering self-efficacy and their beliefs about parental roles directly predicted father involvement in parenting. Maternal gate-opening had both direct and indirect associations with father involvement *via* fathers’ beliefs and fathering self-efficacy. Maternal gate-closing was not associated with father involvement.

**Discussion:**

The findings suggest that fathers’ beliefs about parental roles, fathering self-efficacy, and maternal gate-opening are likely to play an important role in facilitating father involvement with their children. Family interventions and programs could target these modifiable factors to facilitate father involvement in Mainland China.

## 1 Introduction

Fathers play a significant role in the lives of their children (Sarkadi et al., 2008). Fathers’ active and regular involvement with their children has been shown to result in fewer behavioral problems (Amato and Rivera, 1999; Zhang et al., 2019), better academic achievement (Jeynes, 2015; Kim and Hill, 2015), and social-emotional competence (Torres et al., 2014; McMunn et al., 2017) in children. Recent research with Chinese samples indicates similar patterns. For instance, a Chinese study of 1,043 10th-graders in Taiwan showed that increased father involvement significantly predicted lower externalizing and internalizing problem behaviors and higher academic achievement and self-esteem in children (Su et al., 2017). Despite research demonstrating the positive influence of fathers on child adjustment, father involvement with their children is generally lower compared to mother involvement (Baxter and Smart, 2011; Kotila et al., 2013). Father involvement may be even lower in Mainland China due to traditional beliefs about women being responsible for the home. A survey of 6,754 families with children aged 0–5 years in China found that 38.3% of fathers spent less than 1 h per day with their child, compared to 18.9% of mothers (National Health and Family Planning Commission of the People’s Republic of China, 2015). Given the importance of father involvement, it is necessary to examine the factors that may facilitate or hinder Chinese father involvement in parenting.

Father involvement is a multifaceted concept wherein fathers can play a role in parenting in many ways. Lamb et al. (1985) conceptualized father (paternal) involvement as comprising three components: interaction, accessibility, and responsibility. Interaction, also referred to as engagement, involves fathers being directly and actively engaged in activities with their children, such as taking care of them or playing games with them. Accessibility, on the other hand, does not require active interaction and refers to fathers being present and available for their child. For example, a father who is in a different room to the child is said to be accessible to the child as he is available to the child if needed. Finally, responsibility means that fathers take ownership or are accountable for their child’s care and welfare, such as making childcare arrangements and providing financial support and necessities for the child. Lamb et al.’s (1985) tripartite model has been widely used in studies of father involvement (e.g., McBride et al., 2005; Jacobs and Kelley, 2006).

Over the last three decades, research has increasingly focused on fatherhood (e.g., Flouri et al., 2016; McMunn et al., 2017). However, much of this research has predominantly been based on populations in Western countries, and empirical research on Chinese fatherhood is scarce (Chen, 2013; Kwok and Li, 2015; Liu et al., 2016). Parents’ behaviors and beliefs are influenced by their social and cultural environment (Haslam and Mejia, 2017). Chinese traditional cultures, including Confucian, Taoist, and Buddhist philosophies and the patriarchal tradition, exert a strong influence on Chinese fathers’ beliefs and behaviors. Traditional proverbs such as, “Rearing without teaching is the father’s fault” (养不教, 父之过) suggest Chinese fathers’ primary responsibility for educating and disciplining children, whereas childcare was assumed as the responsibility of mothers because of the traditional gender role differentiation (Li and Lamb, 2013). As such, a traditional Chinese father would rarely get involved in everyday childcare. However, under the impact of socio-cultural and economic changes over the last few decades, today’s Chinese fatherhood cannot be simply explained by past culture or traditions. For example, gender equality legislation has changed the status of Chinese men and women both in the family and society (Xu, 2016). The influence of globalization has brought Western values and ideologies into Chinese society. The modernization of Chinese society might have changed the beliefs of Chinese fathers about parenting, which may have increased their involvement with children. Under contemporary Chinese culture, empirical research is needed to examine which factors influence father involvement with their children.

### 1.1 Predictors of father involvement

Although Chinese traditional culture and socioeconomic changes are likely to exert a unique force on Chinese fathers’ beliefs and behaviors, there may be “universal principles” in fatherhood across cultural contexts (Haslam and Mejia, 2017). The model of Chinese father involvement examined in the current study is therefore informed by extant fathering literature from both Western and Asian cultures.

Previous research has examined the relationship between family socio-demographic variables and levels of father involvement with their children. Fathers who are older (Kwok et al., 2012), have higher education (Flouri et al., 2016), and work fewer hours (Bonney et al., 1999; Gaunt, 2006) tend to be more involved in parenting their children. Father involvement has also been found to be higher in families where mothers work a greater number of hours outside the home (Bonney et al., 1999; McBride et al., 2005) and where there is a higher family income (Flouri et al., 2016).

While socio-demographic factors are likely to play a distal role in father involvement, parental process variables, such as father self-efficacy and beliefs, are likely to play a stronger role and potentially reduce the influence of socio-demographic barriers. Freeman et al. (2008) found that father efficacy and beliefs decreased the impact of barriers, such as lack of time and resources and work constraints, on father involvement. That is, a father who believes it is important that he plays an active role in raising his children may make great efforts to engage daily with his children even if he works long hours. Consistent with this, the Hoover-Dempsey and Sandler (1995, 1997) model identified three major constructs essential to parents’ involvement in children’s education and also potentially subject to change through programs and intervention, including parents’ construction of the parental role, parents’ sense of self-efficacy for helping children succeed in school, and general invitations, demands, and opportunities for parental involvement. Parental role construction indicates whether parents deem involvement in children’s education as important and necessary. A high sense of efficacy suggests parents’ strong beliefs in their capabilities to help children succeed in school. These two factors can help to form parents’ motivation to be involved in children’s education. In addition, invitations, demands, and opportunities can create a welcoming and proactive environment to elicit parents’ involvement (Hoover-Dempsey and Sandler, 1995, 1997). Although this parental involvement process model focuses on the mechanisms for parents’ involvement in children’s education, it is argued that these processes can be applied to parental involvement more generally, since education involvement is a central domain of father involvement, particularly for Chinese parents, given their strong emphasis on children’s academic achievement (Pearson and Rao, 2003). Therefore, in our proposed model, the three constructs in Hoover-Dempsey and Sandler’s (1995) model were adapted to beliefs about parental roles, fathering self-efficacy, and maternal gatekeeping to predict Chinese father involvement.

#### 1.1.1 Beliefs about parental roles

Fathers’ beliefs about parental roles identify a range of activities that fathers consider as important and necessary in the care of children (Hoover-Dempsey and Sandler, 1995, 1997). Fathers with non-traditional beliefs toward paternal roles, that is, believing that a father should be more involved in parenting, tended to be more involved in childrearing (Jacobs and Kelley, 2006; Freeman et al., 2008; Schoppe-Sullivan et al., 2013). In the Chinese context, a survey of 2,029 Hong Kong fathers with preschoolers aged 2–6 years found that fathers who strongly believed in the important roles fathers play in child development and had higher fathering self-efficacy were more involved with their children (Kwok and Li, 2015).

#### 1.1.2 Fathering self-efficacy

Fathering self-efficacy can be construed as a father’s beliefs regarding his capability to execute childrearing tasks (de Montigny and Lacharité, 2005). Fathers with a higher sense of parenting efficacy will tend to believe in their capability in this domain; thus, they are likely to be actively engaged in childrearing and put more effort and persevere in the face of challenges that may emerge in parenting (Bandura, 1977; Hoover-Dempsey and Sandler, 1995, 1997). In contrast, fathers who have low levels of efficacy in parenting tend to give up easily and even avoid childrearing tasks. Western research has demonstrated a positive relationship between fathering self-efficacy and father involvement (Jacobs and Kelley, 2006; Freeman et al., 2008; Trahan, 2018). In the Chinese context, fathering self-efficacy is impacted by their children’s achievements and reputation, such as children’s academic achievement and good manners, which symbolize fathering success (Yeh and Yang, 1997; Kwok et al., 2012). Chinese fathers with high parenting self-efficacy externally validated by their children’s achievement and reputation can be more motivated to be involved in parenting to ensure continued fathering success. The previous Chinese study also found that higher fathering self-efficacy is associated with greater father involvement (Kwok and Li, 2015).

#### 1.1.3 Maternal gatekeeping

Mothers can be important in providing fathers with opportunities and engaging them in parenting. The term “maternal gatekeeping” is used to describe the behaviors mothers employ to regulate father involvement, including “gate-opening” and “gate-closing” behaviors (Cannon et al., 2008; Schoppe-Sullivan et al., 2008; Trinder, 2008). Gate-opening behaviors include mothers encouraging father involvement in childrearing and creating opportunities for fathers to interact with their children; gate-closing behaviors refer to mothers discouraging father involvement by criticizing their parenting behaviors or limiting their opportunities to care for their children (Cannon et al., 2008). The relationship between maternal gatekeeping and father involvement has been demonstrated in both Western and Chinese research. Greater maternal encouragement was related to higher levels of father involvement (Schoppe-Sullivan et al., 2008; Fagan and Cherson, 2017; Wang et al., 2019; Zou et al., 2019). Maternal gate-closing was negatively related to the level of father involvement with their children (Fagan and Barnett, 2003; Zou et al., 2019).

One controversy over the model of gatekeeping is the relationship between gate-opening and gate-closing behaviors. Some researchers have conceptualized maternal gatekeeping as lying on a continuum, with one end representing encouragement and the other end representing discouragement (Fagan and Barnett, 2003; McBride et al., 2005; Austin et al., 2013). However, Puhlman and Pasley (2013) suggested treating encouragement and discouragement as separate and independent constructs that can co-occur. For instance, a mother who encourages her child’s father to spend more time with the child may also often criticize the father’s parenting behaviors. Considering the uncertainty of maternal gatekeeping structure, the present study explored the dimension of gatekeeping before analyzing its relationship to father involvement. If there is a significantly negative relationship between gate-opening and gate-closing, they will be combined to represent gatekeeping; if not, they will be treated as two independent components in the analysis.

Apart from a direct influence, we speculate that maternal gatekeeping can exert an indirect effect on father involvement by influencing fathers’ beliefs about parental roles and their parenting self-efficacy. Verbal persuasion is seen as one source of self-efficacy beliefs (Bandura, 1977). When people are persuaded that they are able to cope successfully with difficult situations, they are likely to initiate greater efforts in the face of adversity. Accordingly, mothers’ encouragement and praise can lead fathers into believing that they are capable of organizing and executing childrearing tasks, which in turn increases father involvement. In contrast, mothers’ discouragement and criticism might make fathers doubt their capability and decrease their self-efficacy for coping with difficulties in parenting, which subsequently decreases the levels of father involvement. Similarly, maternal gate-opening and gate-closing might validate or change fathers’ beliefs about the importance and benefits of father involvement, thus indirectly affecting the levels of fathers’ involvement with their children. Indeed, Kwok and Li (2015) found that Chinese fathers’ role beliefs and parenting self-efficacy mediated the relationship between spousal capital, including parenting alliance, marital satisfaction, and spousal support, and their involvement with children, though this study focused more on the influence of couple and co-parenting relationship on father involvement. Therefore, it is likely that maternal gatekeeping has an indirect impact on father involvement *via* fathers’ beliefs and fathering self-efficacy, though empirical research is needed to test this hypothesis.

### 1.2 The present study

The current study used a cross-sectional survey of Chinese mother-father dyads to test our proposed model of father involvement in Mainland China. The review of the literature suggested that father involvement is the product of a variety of factors. However, based on the model of Hoover-Dempsey and Sandler (1995), we focused on parental process variables, including fathers’ beliefs about parental roles, fathering self-efficacy, and maternal gatekeeping. In the proposed model, we hypothesized that (a) fathers’ non-traditional beliefs about parental roles and higher fathering self-efficacy, and mothers’ greater usage of gate-opening and lower usage of gate-closing behaviors are directly associated with greater father involvement in parenting; and (b) mothers’ greater usage of gate-opening and lower usage of gate-closing behaviors are associated with fathers having more non-traditional beliefs about parental roles and higher fathering self-efficacy, which is in turn associated with fathers being more involved in parenting.

The participants of this study were Chinese parents of preschoolers. Children experience rapid development and growth during the preschool years, and parental involvement is very important. However, traditional Chinese fathers are less involved in parenting until children reach the “age of reason” (around the time children start school). The focus on father involvement during the preschool years provided the opportunity to explore the predictors of contemporary Chinese father involvement when their children are young.

## 2 Materials and methods

### 2.1 Participants

The final sample comprised 609 Chinese couples with at least one child in preschool. The survey was returned by 1,092 families but the data of 483 couples were excluded because at least one parent in the couple answered less than 50% of the survey.

The mean age of children was 4.61 (SD = 0.99). Child gender was equally represented (50.7% were male), and just over half of families (56.8%) reported having only one child. Fathers’ mean age was 35.05 (SD = 4.39), while that of mothers was 33.14 (SD = 4.01). Around half of fathers (52.8%) and mothers (49.3%) had a bachelor or higher degree, and the majority of them (94.4% of fathers and 78.2% of mothers) were employed. The mean working hours were 39.92 (SD = 19.16) for fathers and 28.64 (SD = 19.79) for mothers. Most parents (99.0%) were married, and around two-thirds of them (64.0%) lived with their own parents (the child’s grandparents). Around half (55.9%) of families had a monthly family income over ¥8,000 (approximately USD 1,130). Most families (88.3%) could meet their essential expenses, and about half of those (45.0%) had enough money to purchase most of the things they really wanted.

### 2.2 Procedure

Ethical approval was obtained from the authors’ university ethics committee. The study used convenience sampling. Eleven preschools were contacted (four public and seven private preschools) in Fuzhou city (7.115 million people), which is the capital of Fujian province and is located on the southeast coast of Mainland China. Preschool teachers introduced the survey to parents when they picked up their children. Interested parents received a survey package with an information sheet, two questionnaire packs (one for mother, another for father), and two small envelopes. The information sheet instructed parents to complete the survey independently of their partner, to seal the questionnaire in a small envelope, and then to replace the two sealed envelopes into the survey package. The parents were encouraged to return the completed questionnaire to their child’s teacher within 1 week. In total, 1,610 survey packages were delivered to parents, and 1,092 (68%) couples returned the survey.

### 2.3 Measures

#### 2.3.1 Demographic information

The Family Background Questionnaire (FBQ; Sanders and Morawska, 2010) was used to collect demographic information. The questionnaire asks about parent and child age and gender, parent marital status, work and education, family composition, and financial situation. Mothers reported demographic information related to themselves and their families, and fathers reported their own demographic information.

#### 2.3.2 Father involvement

Fathers reported their own level of involvement with their child using the Father Involvement Questionnaire (Wu et al., 2015). This 56-item questionnaire assesses three dimensions of father involvement, including interaction (22 items; e.g., “I play with my child at home”), accessibility (8 items; e.g., “Even if I do my own things at home, I pay attention to my child’s needs”), and responsibility (26 items; e.g., “I financially support my child’s development”). The measure has been validated in a sample of Chinese fathers (Wu et al., 2015). Fathers rated how often they engage in various parenting behaviors on a 5-point scale, ranging from 0 (Never) to 4 (Always). The items were averaged to yield summary scores for the three domains of parental involvement, with higher scores indicating higher levels of parental involvement with their child. Cronbach’s alphas of the three subscales in the present study were 0.94, 0.86, and 0.94, respectively.

#### 2.3.3 Fathering self-efficacy

Fathers reported their self-efficacy or confidence to deal with their child’s behavioral and emotional problems using the 19-item self-efficacy subscale of the Child Adjustment and Parent Efficacy Scale (CAPES; Morawska et al., 2014). Fathers rated their confidence in managing their child’s problems (e.g., “My child loses their temper”) on a 10-point scale, ranging from 1 (Certain I can’t do it) to 10 (Certain I can do it). Items were averaged to yield an efficacy score, with higher scores indicating that fathers have a higher level of self-efficacy in parenting. The reliability coefficient for the present study was 0.96.

#### 2.3.4 Fathers’ beliefs about parental roles

Fathers reported their beliefs about parental roles in child care using the 26-item Beliefs Concerning the Parental Role Scale (BCPR; Bonney and Kelley, 1996). They rated their agreement with items related to the mother’s role (e.g., “It is mainly the mother’s responsibility to change diapers”) and the father’s role [e.g., “It is important for a father to spend quality time (one-to-one) with his children every day”] on a 5-point scale, ranging from 1 (Agree strongly) to 5 (Disagree strongly). Items were averaged to yield a summary score, with higher scores indicating more liberal beliefs concerning parental roles; that is, believing that the father should be more involved in parenting. In the present study, due to negative factor loadings in the confirmatory factor analysis (CFA) using the present sample, two items (items 17 and 25) were removed from further analysis. Cronbach’s alphas for the present study were 0.87.

#### 2.3.5 Maternal gatekeeping

Both parents completed an adapted version of the Parental Regulation Inventory (PRI; Van Egeren, 2000) to report on their perceptions of maternal gate-opening (9 items) and gate-closing behavior (9 items). Mothers rated how often they responded to the parenting behaviors of the child’s father with gate-opening behavior (e.g., “Compliment your baby’s father”) or gate-closing behavior (e.g., “Look exasperated and roll your eyes”) on a 6-point scale, ranging from 1 (Never) to 6 (Several times a day/Every time). Fathers rated the frequency that their child’s mother engaged in these same gatekeeping behaviors. The items of gate-opening and gate-closing were averaged separately for mothers and fathers to yield summary scores on each subscale. In the present study, the reliability coefficients for gate-opening were 0.89 for mothers and 0.90 for fathers, and those for gate-closing were 0.86 for mothers and 0.89 for fathers.

### 2.4 Missing data

In the entire dataset, 3,423 out of 207,669 data (1.65%) were missing. The largest proportion of missing data came from father age where 21.2% of data were missing. Little’s missing completely at random (MCAR) test, conducted both on the mother dataset, χ^2^ (50,981) = 53,582.64, *p* < 0.01, and on the father data set, χ^2^ (32,809) = 34,809.50, *p* < 0.01, showed that the two datasets were not MCAR. A dummy variable with two values was created to represent missing and non-missing cases, and logistic regressions were performed to identify associations between the observed variables and missingness. The missingness of the two datasets was found to be related to the auxiliary variables (e.g., father’s and mother’s education and father’s working hours), so the two datasets were presumed missing at random (MAR). Thus, expectation maximization algorithms were used to impute missing values.

### 2.5 Data analysis

To verify the presumed dimensionality of the measures, CFA was conducted for each measure before further data analysis (Little et al., 2002). Since the purpose of this study was to examine relationships among multiple constructs rather than scale validation, the items of the constructs were parceled to indicate latent variables (Matsunaga, 2008). The method of *item-to-construct balance* was adopted to create three parcels for fathering self-efficacy and fathers’ beliefs about parental roles, since the two measures were unidimensional and used established scales (Little et al., 2002). Construct validation of the Father Involvement Questionnaire in Chinese fathers suggested the three dimensions of father involvement (Wu et al., 2015), so the items in each subscale were averaged to create an indicator for the latent factor of father involvement based on the *internal-consistency approach* (Kishton and Widaman, 1994). Mothers’ and fathers’ reports of maternal gate-opening (*r* = 0.41, *p* < 0.01) and gate-closing (*r* = 0.34, *p* < 0.01) were significantly correlated. Since both parents provided valuable information about the perceptions of mothers’ behaviors, the 9 items of fathers’ and mothers’ reports of maternal gate-opening were averaged separately and used as two indicators of the latent variable of gate-opening. The same process was used for the latent variable of gate-closing.

Descriptive analyses were conducted using SPSS 24.0. To examine the structural relationships of the proposed models, structural equation modeling (SEM) was performed in Mplus 8.1 (Muthén and Muthén, 2018). Before examining the fit of the proposed structural equation models, the fit of the measurement models was tested using CFA. Several indices were used to evaluate model fit, including the chi-square (χ^2^) index, comparative fit index (CFI), Tucker–Lewis index (TLI), root mean square error of approximation (RMSEA), and the standardized root mean squared residual (SRMR). Comparative fit index and TLI above 0.90, RMSEA below 0.06, and SRMR below 0.08 suggested a relatively good model fit (Hu and Bentler, 1999). To examine the indirect path between gatekeeping and father involvement, the bias-corrected bootstrapping procedure with 1,000 resamples was conducted to yield a 95% confidence interval (CI). If the 95% CI did not contain zero, this suggested significant indirect effects (Williams and Mackinnon, 2008).

## 3 Results

The correlations between the demographic variables and the three dimensions of father involvement were shown in Table 1. Fathers had higher levels of involvement in parenting on at least one dimension, when parents were older, employed, and had higher levels of education. Mothers’ working hours, but not fathers’ working hours, were significantly and positively related to father interaction with children. In addition, fathers were more engaged in childrearing, when their wives believed that they have enough money to purchase most of the things they really want. As parents’ age, education, employment status, mothers’ working hours, and family financial situation were significantly associated with at least one dimension of father involvement, these demographic factors were included in the structural equation models as the controlled variables.

**TABLE 1:** Correlations of dimensions of father involvement with demographic variables.

Demographic variables	Father interaction	Father accessibility	Father responsibility
Child gender	0.00	–0.02	0.04
Child age	0.07	0.05	0.06
Number of children^a^	–0.03	–0.02	0.00
Father age	0.08*	0.07	0.08*
Mother age	0.08	0.06	0.10*
Father education^a^	0.23*	0.15*	0.20*
Mother education^a^	0.12*	0.06	0.09*
Father employment	–0.08	−0.08*	−0.12**
Mother employment	−0.11**	–0.03	–0.06
Father working hours	–0.05	0.02	0.05
Mother working hours	0.11**	0.04	0.06
Lived with grandparents	–0.01	–0.01	–0.00
Monthly family income^a^	0.06	0.05	0.05
After paid for essential expenses^a^	−0.18**	−0.18**	−0.15**

Child gender: 1 = Male, 2 = Female; Father and mother education: 1 = Middle school or less, 2 = High school, 3 = College certificate, 4 = Bachelor degree, 5 = Master degree or higher; Father and mother employment: 1 = Employed, 2 = Not employed; Lived with grandparents: 0 = No, 1 = Yes; Monthly family income: 1 = Less than ¥3,999, 2 = ¥4,000–¥7,999, 3 = ¥8,000–¥11,999, 4 = ¥12,000–¥15,999, 5 = ¥16,000–¥19,999, 6 = ¥20,000–¥23,999, 7 = More than ¥24,000; After paid for essential expenses: 1 = Enough to purchase most of the things, 2 = Enough to purchase some of the things, 3 = Not enough to purchase much of anything. ^a^Correlations with these variables are Spearman rho coefficients. *p < 0.05 and **p < 0.01.

Table 2 shows the correlations among fathers’ and mothers’ beliefs about parenting, maternal gatekeeping, and father involvement. Fathers were more involved with their children on all three dimensions when they reported non-traditional beliefs about parental roles and higher fathering self-efficacy. Higher levels of father involvement were related to mothers and fathers reported higher levels of maternal gate-opening behavior, but not maternal gate-closing behavior.

**TABLE 2:** Correlations, means and standard deviations of father involvement, fathers’ beliefs about parental roles, fathering self-efficacy, and maternal gatekeeping.

Variable	*M* (SD)	1	2	3	4	5	6	7	8
1. Father interaction	2.42 (0.65)	–							
2. Father accessibility	2.65 (0.73)	0.79**	–						
3. Father responsibility	2.56 (0.64)	0.85**	0.81**	–					
4. Fathers’ beliefs	3.61 (0.53)	0.37**	0.41**	0.36**	–				
5. Fathering self-efficacy	7.23 (1.78)	0.39**	0.34**	0.39**	0.25**	–			
6. Gate-opening (M)	3.81 (1.03)	0.31**	0.25**	0.29**	0.16**	0.16**	–		
7. Gate-closing (M)	3.34 (0.94)	0.03	0.02	0.01	–0.07	−0.09*	0.13**	–	
8. Gate-opening (F)	3.74 (1.03)	0.34**	0.28**	0.36**	0.20**	0.18**	0.41**	0.06	–
9. Gate-closing (F)	3.41 (1.00)	0.03	0.06	0.04	−0.12**	–0.07	0.05	0.34**	0.24**

M, mother report; F, father report. *p < 0.05 and **p < 0.01.

As the correlations between maternal gate-opening and gate-closing were positive, suggesting the coexistence of gate-opening and gate-closing behavior, two models were tested to examine the role of maternal gate-opening (Model One) and maternal gate-closing (Model Two) as predictors of father involvement separately.

Two measurement models were tested first. The first model included four latent variables representing father involvement, fathering self-efficacy, fathers’ beliefs about parental roles, and maternal gate-opening, and all variables were allowed to correlate with each other. The model fitted the data well, χ^2^ (38) = 69.19, *p* = 0.002; RMSEA = 0.04; 90% CI [0.02, 0.05]; CFI = 0.99; TLI = 0.99; and SRMR = 0.02. All factor loadings were significant and ranged from 0.60 to 0.95. The second model replaced maternal gate-opening in the first model with maternal gate-closing, but kept the other three latent variables. The model fitted the data well, χ^2^ (38) = 61.32, *p* = 0.010; RMSEA = 0.03; 90% CI [0.02, 0.05]; CFI = 1.00; TLI = 0.99; and SRMR = 0.02. All factor loadings were significant and ranged from 0.49 to 0.95.

**FIGURE 1:**
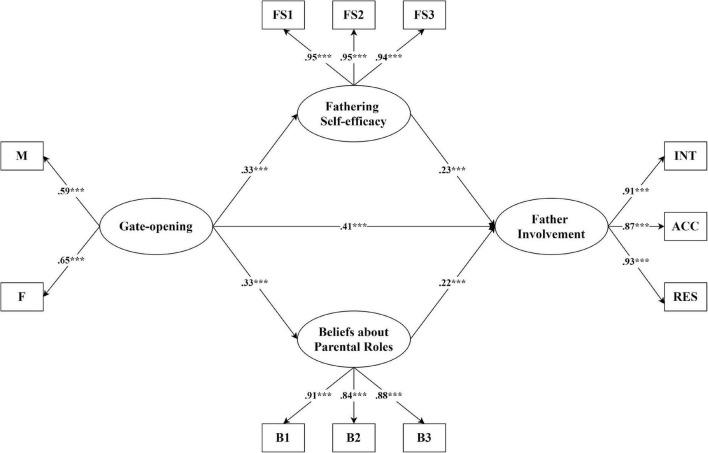
Structural equation model of relationships among fathering efficacy, fathers’ beliefs, gate-opening, and father involvement. Estimates are presented in standardized units. Model fit: χ^2^ (103) = 198.66, *p* < 0.001; RMSEA = 0.04; 90% CI [0.03, 0.05]; CFI = 0.98; TLI = 0.97; and SRMR = 0.04. ***p < 0.001.

In the model testing maternal gate-opening behavior as one of the predictors of father involvement (see Figure 1), the results indicated that the model fitted the data well, χ^2^ (103) = 198.66, *p* < 0.001; RMSEA = 0.04; 90% CI [0.03, 0.05]; CFI = 0.98; TLI = 0.97; and SRMR = 0.04. After controlling for the demographic variables, fathers’ beliefs about parental roles (β = 0.22, *p* < 0.001), fathering self-efficacy (β= 0.23, *p* < 0.001), and maternal gate-opening (β = 0.41, *p* < 0.001) were three significant predictors of father involvement. Maternal gate-opening also significantly predicted fathers’ beliefs about parental roles (β = 0.33, *p* < 0.001) and fathering self-efficacy (β = 0.33, *p* < 0.001), and had a significant and indirect impact on father involvement *via* fathers’ beliefs (β = 0.07, *p* < 0.001) and fathering self-efficacy (β = 0.08, *p* = 0.001). In total, this model explained 47.4% of the variance of father involvement.

**FIGURE 2:**
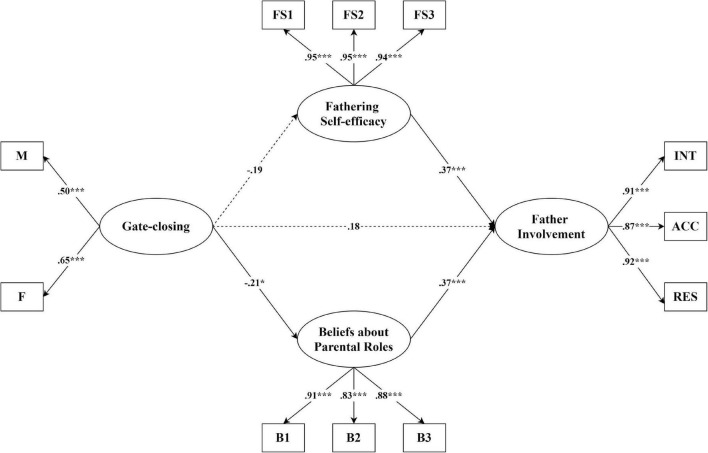
Structural equation model of relationships among fathering efficacy, fathers’ beliefs, gate-closing, and father involvement. Estimates are presented in standardized units. Model fit: χ^2^ (103) = 190.54, *p* < 0.001; RMSEA = 0.04; 90% CI [0.03, 0.05]; CFI = 0.98; TLI = 0.98; and SRMR = 0.05. *p < 0.05, ***p < 0.001.

In the model testing maternal gate-closing behavior as one of the predictors of father involvement (Figure 2), the pattern of results was different from those in the model with gate-opening behavior included. The model fitted the data well, χ^2^ (103) = 190.54, *p* < 0.001; RMSEA = 0.04; 90% CI [0.03, 0.05]; CFI = 0.98; TLI = 0.98; and SRMR = 0.05. As in the previous model, fathers’ beliefs about parental roles (β = 0.37, *p* < 0.001) and fathering self-efficacy (β = 0.37, *p* < 0.001) were direct predictors of father involvement, after controlling for the demographic variables. Maternal gate-closing, however, was not significantly associated with father involvement either directly or indirectly, although it did negatively predict fathers’ beliefs about parental roles (β = −0.21, *p* = 0.043). In total, the whole model explained 34.7% of the variance of father involvement.

## 4 Discussion

The aim of the present study was to identify the predictors of father involvement in Mainland China. Based on Hoover-Dempsey and Sandler’s (1995) model of the parental involvement process, fathers’ beliefs about parental roles, fathering self-efficacy, and maternal gatekeeping were included in the proposed model for analysis. The results supported the hypotheses about the relationship between fathers’ beliefs, fathering self-efficacy, and father involvement. Fathers who had more liberal beliefs about parental roles and had higher self-efficacy were more likely to be involved in childrearing. These findings are similar to previous research on father involvement conducted in the US (Freeman et al., 2008) and China (Kwok and Li, 2015), which may suggest the consistency in relations of fathers’ beliefs about parental roles and fathering self-efficacy to father involvement across countries and cultures.

Concerning the role of maternal gatekeeping, however, the findings only partially supported the hypotheses. Maternal gate-opening had both direct and indirect associations with father involvement *via* fathers’ beliefs and fathering self-efficacy. Gate-closing, however, had no significant direct or indirect impact on father involvement. Although previous research indicated the direct relation between maternal gate-opening and father involvement in Chinese families (Wang et al., 2019; Zou et al., 2019), the present study also suggested the indirect association among parents’ beliefs and behaviors. That is, mothers’ greater use of gate-opening behaviors was associated with fathers having more liberal beliefs about parenting roles and with fathers having greater confidence in their own parenting, which in turn were associated with fathers being more involved in parenting. Thus, it is possible that fathers’ commitment to their parental role can be affirmed by their partner’s encouragement, leading to a change in fathers’ behavior - an increase in father involvement. The significant relation between maternal gate-opening and father involvement is also a prime example of the interdependence of individuals’ behaviors and relationships in the family (Minuchin, 1985) and underscore the importance of evaluating Chinese fathers’ behavior in the context of the family system (Schoppe-Sullivan et al., 2015).

Maternal gate-closing, however, was not directly or indirectly significantly associated with father involvement. Similar results were also found in the US study, which used the same questionnaire to measure maternal gatekeeping, and failed to find a significant relationship between gate-closing and father involvement (Schoppe-Sullivan et al., 2008). Yet, in two Chinese studies, maternal discouraging behaviors were significantly associated with father involvement (Wang et al., 2019; Zou et al., 2019). Two possible reasons might explain this inconsistency in the findings. Firstly, the data regarding maternal gatekeeping in these four studies were collected from different respondents. The two Chinese studies were only based on mothers’ responses, whereas the present and the US study used both parents’ reports. Perceptions of fathers and mothers could be different, which may influence the results. Secondly, some items (e.g., “Tell your baby’s father the right way to handle the situation”) in the measure of maternal gate-closing, used in both the present and the US study, may not be identified as criticism or discouragement for some fathers. For example, a mother’s words, “You need to leave him alone till he calms down,” can be perceived as criticism or helpful reminder by the father depending on the mother’s tone (e.g., angry or supportive) and the father’s perception and cognition. Thus, further research can reconsider the validity of the measure of maternal gate-closing to see whether these items represent maternal criticism or discouragement before examining the relationship between maternal gate-closing and father involvement.

Although parents’ age, education, employment status, mothers’ working hours, and family financial situation were significantly associated with father involvement, fathers’ beliefs about parental roles, fathering self-efficacy, and maternal gate-opening were important contributing factors of father involvement after controlling for these demographic variables. These findings highlighted the stronger roles of parents’ own beliefs and behaviors in influencing father involvement.

The present study was one of the first to identify the predictors of father involvement in Mainland China. Study strengths include the use of multiple-informant approach, a large sample size, and a good participation rate, especially considering the low participation of fathers in family-based research (Cassano et al., 2006). This research design provided an opportunity to explore the possible mechanisms of father involvement in the family context, and considered both parents’ behaviors, beliefs, and perceptions.

### 4.1 Limitations and future directions

There are several limitations that should be acknowledged. Firstly, as a cross-sectional survey, causal relationships among variables cannot be established and it is impossible to determine the direction of influence between mothers and fathers, and whether the relationship between maternal gate-opening and fathers’ beliefs and behaviors is a pathway of influence beginning from the mother, from the father, or, what is more likely, a reciprocal relationship between parents. Further longitudinal or experimental research is necessary to explore the direction of relationships among parents’ beliefs and behaviors and to establish causal relationships among them.

Secondly, the sample was restricted to Chinese couples in urban areas, most of whom were married, highly educated, and middle class. Considering the rural-urban disparity in China, the sampling may limit the generalisability of the findings, especially for applying those findings to parents in rural areas. Further studies should use stratified sampling and include Chinese families from different regions and both urban and rural areas. More attention should also be paid to special and vulnerable groups, such as ethnic minorities, single-parent families, migrant families, and families with left-behind children. These fathers may face unique and specific challenges and difficulties in their involvement with children.

Thirdly, the present study only focused on mothers’ influence on fathers’ beliefs and behaviors. Yet, the demographic information indicated that 64% of grandparents lived with parents. Grandparents may also play a role in parenting, and their beliefs and behaviors may influence father involvement. Further research should consider the influence of other family members (e.g., grandparents) on fathers’ behaviors and beliefs. Is it possible that grandparents also play a gate-opening or gate-closing role in influencing father involvement?

### 4.2 Conclusion and implications

Our findings indicated that fathers’ beliefs about parental roles, fathering self-efficacy, and maternal gate-opening were significant predictors of father involvement, which suggested the importance of changing parents’ behaviors and beliefs to facilitate father involvement in parenting. Under the impact of socio-cultural and economic changes (e.g., gender equality and the influence of globalization), Chinese parents’ beliefs about parental roles might also change, which might gradually influence how Chinese fathers behave in parenting. However, it does not mean we should wait there and rely on gradual socio-cultural and economic changes to influence fathers’ beliefs and behaviors. Fathers’ beliefs about parental roles, fathering self-efficacy, and maternal gate-opening are all dynamic and modifiable factors. To facilitate father involvement, family interventions, and programs could target fathers’ beliefs about parental roles, fathering self-efficacy, and help parents learn to encourage each other in parenting to strengthen fathers’ commitment to parental roles.

## Data availability statement

The raw data supporting the conclusions of this article will be made available by the authors, without undue reservation.

## Ethics statement

The studies involving human participants were reviewed and approved by the University of Queensland School of Psychology Ethics Committee. The patients/participants provided their written informed consent to participate in this study.

## Author contributions

YL: conception and design, participant recruitment and data collection, data analysis and interpretation, and manuscript writing. DH and CD: conception and design, data analysis and interpretation, and manuscript editing. MG: conception and design, participant recruitment and data collection, data analysis and interpretation, and manuscript editing. AM: data analysis and interpretation and manuscript editing. All authors contributed to the manuscript and approved the submitted version.
